# Experiences of decision making about psychotropic medication during pregnancy and breastfeeding in women living with severe mental illness: a qualitative study

**DOI:** 10.1007/s00737-023-01325-0

**Published:** 2023-05-12

**Authors:** J. Frayne, R. Ellies, T. Nguyen

**Affiliations:** 1 Medical School, Discipline of General Practice, The University of Western Australia, Crawley, Australia; 2 Department of Obstetrics, Women and Newborn Health Service, Subiaco, Western Australia Australia; 3 Peel and Rockingham Kwinana Mental Health Services, Rockingham, Australia; 4 Medical School, Discipline of Psychiatry, The University of Western Australia, Crawley, Australia

**Keywords:** Patient perspectives, Psychosis, Bipolar, Pregnancy, Breastfeeding, Psychotropic medication

## Abstract

**Purpose:**

The aim of this study was to explore the experiences of women living with severe mental illnesses making decisions about psychotropic medication use in pregnancy and breastfeeding, and what helped or hindered the decision-making process.

**Methods:**

We report on a qualitative study from 12 women who attended the pregnancy service between May 2018 and June 2019. Interviews occurred at 4–6 weeks postpartum on women with severe mental illnesses, which was nested within a larger mixed-methods study.

**Results:**

Three main themes were elicited from the participants’ transcriptions and included (i) the decision-making process with subthemes of shared decision-making, consistency and complete care, collaboration and clear communication, and challenges of managing medication; (ii) how information is given, with subthemes of information delivery and communication breakdown; and (iii) breastfeeding dilemmas with subthemes of lithium and breastfeeding choice and autonomy regarding breastfeeding on medication.

**Conclusion:**

Findings offer understanding of patients’ experiences in the decision-making and use of psychotropic medication during pregnancy and breastfeeding. Women living with severe mental illnesses, such as bipolar and psychosis, face difficult medication decisions due to uncertainty around use in pregnancy, potentially causing conflict with their dual role as both persons with a diagnosed mental illness but also new mothers. The clinician needs to provide comprehensible and concise information, giving space for a woman’s voice to be heard to guide them from a position of hesitancy to one of assurance. Collaboration within a multidisciplinary team and external care providers combined with consistency of care assists this process.

**Supplementary Information:**

The online version contains supplementary material available at 10.1007/s00737-023-01325-0.

## Introduction

Many women with severe mental illnesses (SMI) are mothers (Howard et al. 2001), and in Australia, over half of women with an SMI diagnosis have had children (Morgan et al. 2014). SMI can have long-term impact on the well-being of mother and child due to a constellation of risk factors, including the severity of the mental illness and the challenges associated with its treatment, and this is especially so for those living with severe mental illness defined in this study as those with schizophrenia and other psychosis, and bipolar disorders (Jones et al. 2014). Over recent decades, the prescribing of fertility-sparing medication increased access to specialized perinatal mental health services, and support in their parenting choices (Nicholson 2014) has seen women living with SMI become mothers; therefore, managing their pregnancy care is crucial to ensuring optimal outcomes. There is a need for health professionals to develop enhanced models of pregnancy care, as well as act collaboratively, considering the patients’ and their families’ perspectives (Kim 2012; Shah 2012).

Factors that inform management for both parents and treating clinicians include the nature and stability of the illness, uncertainty about relative risks and benefits, the consequences of untreated illness, and exposure to medications for the fetus in the antenatal and postnatal period (Jones et al. 2014). The impact mental illness has on women with SMI in their capacity to make informed decisions, feelings of reduced autonomy over decision-making, and the process and stigma regarding their ability to be caring parents (Bagadia et al. 2020) is an important consideration.

Several qualitative studies have recently been released discussing the experiences of women within perinatal services (Bagadia et al. 2020; Banerjee et al. 2021), but little remains known as to how women’s experiences impact their decisions regarding medication use and care through pregnancy and postnatally, specific for those living with severe mental illness such as bipolar disorders and schizophrenia. A narrative review of decision-making for antidepressant use during pregnancy by Hippman and Balneaves (2018) suggests that women want a nonjudgmental environment where this can occur. Furthermore, they describe barriers and facilitators to this process rely on cognitive factors, including trustworthy sources of information, emotional factors related to severity of illness, stigma and guilt, and social support (Hippman and Balneaves 2018).

To gain a better understanding of psychopharmacological treatment within a specialized perinatal mental health service, we undertook a qualitative study nested within a larger mixed methods study. The aim of this qualitative study was to explore the experiences women living with severe mental illness had in making decisions about psychotropic medication use. Additionally, we wished to explore the information that was provided on medications in pregnancy and breastfeeding, and what helped or hindered women’s decision-making process.

## Method

This nested qualitative study was part of a larger mixed methods naturalistic study (Frayne et al. 2020) involving longitudinal data collected over the course of the pregnancy for women who were seen at an antenatal service dedicated to the management of women with SMI. We followed a pragmatic paradigm to guide our study, which focused on an understanding of the complex needs for pregnant women with SMI and on efforts to provide practical guidance and give meaning to and understand the consequences of our health care delivery. We employed a postpositivist approach with the assumption of an external reality that can be documented and understood (factors that impact women’s perceptions and experiences of taking psychotropic medications during pregnancy and breastfeeding) as well as objective methodology with co-author RE, an independent interviewer.

Diagnoses for the service including schizophrenia or psychosis (ICD code: F20, F25, F28), bipolar affective disorders (F31), and severe non-psychotic condition, with antenatal care delivered through a multidisciplinary team comprising of a psychiatrist, obstetrician, midwife, social worker, and dietician, further described in detail elsewhere (Frayne et al. 2019). The decision-making model developed by Wisner et al. (2000) for risk-benefit discussions around psychotropic medication use in pregnant women is used within this service and is delivered at their first booking appointment (usually between 12- and 20-week gestation) and continuously discussed throughout their pregnancy visits as needed.

Women who attended for care between May 2018 and June 2019 were recruited at their first antenatal appointment into the prospective mixed method study. Permission was obtained to contact them for a postpartum interview. The hospital human research ethics committee approved the study (RGS0000000532), and informed written consent was obtained. Demographic, pregnancy, and mental health data was collected during the pregnancy as per the larger mixed methods study protocol (Frayne et al. 2020). Interviewees were selected sequentially upon their delivery. Telephone interviews, of 15- to 45-min duration, took place between 4 and 6 weeks postpartum. A semi-structured questionnaire was used for all interviews containing predominantly open questions encompassing three overarching topics relating to (i) information, (ii) barriers and facilitators, and (iii) support (see Appendix 1).

Qualitative data were transcribed, imported into NVivo version 12, along with undergoing sequential inductive analysis by RE, who was independent to the healthcare provided. Analytical memos were employed to further explore and reflect on the data. Participants were de-identified and described as P1-12. Codebook thematic analysis occurred after each interview, with data familiarization, coding and generating initial themes using an inductive/deductive method. Codebook themes were generated from reviewing the literature and after an initial read of the raw data by author RE, a psychiatrist, and then by JF, who had provided nonpsychiatric care to the women in pregnancy. Subthemes and themes were identified at a semantic level, conceptualizing ideas from the interviews. All authors developed and reviewed, refined and then defined theme labels (Braun and Clarke 2006). Saturation as described by Ando et al. (2014) and defined in this study as the point at which limited new concepts or descriptions relevant to the research aims were found after 12 recorded interviews.

## Results

A description of the sample of participants is shown in Table 1.

**Table 1:** Demographics, psychosocial, and clinical profile of women with SMI

Patient	Age (years)	Country of birth	^#^Parity	Education level attained	Psychiatric diagnosis	Medication
P1	31	Australia	1	Not completed	Bipolar type 1	lithium 450 mg lamotrigine 50 mg, quetiapine 50 mg
P2	29	Australia	2	Year 12	Bipolar type 1	lithium 1250 mg quetiapine 200 mg
P3	33	Australia	1	Tertiary	Bipolar type 2	lithium 900 mg duloxetine 120 mg escitalopram 20 mg lamotrigine 200 mg quetiapine XR 250 mg
P4	36	Australia	1	Tertiary	Bipolar type 1	aripiprazole 10 mg
P5	30	Australia	2	Year 12	Bipolar type 2	Fluoxetine 40 mg lamotrigine 400 mg quetiapine 100 mg
P6	41	Australia	2	Tertiary	Bipolar type 1	lamotrigine 200 mg escitalopram 10 mg olanzapine 2.5 mg
P7	34	Australia	6	Not completed	Bipolar type 2	Lithium 450 mg Olanzapine 5 mg mirtazapine 30 mg
P8	28	Afghanistan	1	Year 12	Schizophrenia	olanzapine 5 mg
P9	33	Australia	3	Tertiary	Bipolar type 2	quetiapine XR 300 mg
P10	37	Australia	1	Trade certificate	Bipolar type 1	lamotrigine 300 mg sertraline 100 mg
P11	33	Australia	2	Trade certificate	Schizophrenia	aripiprazole 10 mg olanzapine 10 mg
P12	31	Ethiopia	2	Trade certificate	Schizophrenia	aripiprazole depo 400 mg

#parity = births completed

Three main themes were elicited from the participants’ transcriptions and included the decision-making process, information giving, and breastfeeding dilemmas. Table 2 describes these further with the subthemes listed.

**Table 2:** Themes and subthemes on the experiences of women with severe mental illness making decisions about psychotropic medication use

Theme	Subtheme	Description
The decision-making process	Shared-decision making	Giving information and sharing the decision-making process was important for women to understand balancing the risk of medication.
	Consistency and complete care	Developing a holistic approach, feeling listened to, and having consistency in care
	Collaboration and clear communication	Consistent and clear advice across health providers
	The challenges of managing medication	Complicated further by polypharmacy, pregnancy, potential medication change needed
How information is given	Information delivery	Past knowledge and experiences, and how information was presented both verbally and nonverbally
	Communication breakdown	Due to cultural and language barriers being underestimated
Breastfeeding dilemmas	Lithium use and breastfeeding choice	Conflicting pressures
	Autonomy regarding breastfeeding on medication	Patient-centered focus, breastfeeding choices, support

### The decision-making process

#### Shared decision-making

Most women reported a discussion on risk-benefit analysis, with risks of medication exposure to the baby antenatally and postnatally weighed against the risks of destabilizing their mental health. “*[Psychiatrist] went through the side effects …we both decided that it was safest for me and my mental health if I stayed on the same medications* (P5).” A shared decision-making process occurred often with the majority expressing that the clinicians considered their wishes when it came to treatment changes while ensuring they understood the risks involved. This process of deliberation occurred over multiple appointments, “*I did stop the lithium about three weeks before he was born so I could try breastfeeding and I haven’t gone back…. That was my decision, but I had a lot of advice regarding that* (P7).” A patient-centered approach included being heard, respected, and supported in choices. *“I had a good experience, everyone listened to me, took into consideration my feelings and the things that I wanted…* (P7).” When a paternalistic model was used, women felt less engaged with these decisions, “*Not good for baby, so they changed the medication to a different one… They gave me information, but I forgot* (P12).”

#### Consistency and complete care

The health provider-patient interaction women experienced from receiving care through a multidisciplinary team was central to developing a holistic approach. “*You knew that you were seeing your obstetrician, your midwife and your psychiatrist. I felt like I was being looked after head to toe* (P6).” It provided an understanding of how their mental health was interconnected with their antenatal care. “*For me the reason I was there was for mental health issues, and I felt this was really well looked after during pregnancy…’* (P3) and *‘…because my mental health wasn’t great and having a lot of appointments where people listened to me, I felt really, good about that…* (P7).”

All women commented that consistency in staff was a strong factor in developing rapport and trust required to make treatment decisions. Even when care was perceived as being of high quality, “*It was very good, happy with the doctors I saw - but it would have been better to have more consistency* (P4).” Not only consistency during their pregnancy care but having a long-standing therapeutic relationship within the community was valued. “*Important [having the same psychiatrist through pregnancy] - but not as important as I have a psychiatrist outside the hospital that I have been seeing for a long time* (P9)” and “*It was mainly my GP who had gone through that, but additionally I had seen the psychiatrist, which was the same psychiatrist that I had last time [in pregnancy]* (P5).” Women without this consistency expressed difficulties in establishing a strong therapeutic relationship, “*That [psychiatrist] kept swapping and changing, I didn’t know who was there really* (P2).”

#### Collaboration and clear communication

All interviewed women were on psychotropic medication, either with monotherapy or more commonly polytherapy, prior to engagement with the antenatal service. Some were managed in the community with access to community mental health services or private psychiatrists and others by their general practitioners. Collaboration and clear communication with community health providers were seen as important components to care. “*We were linked in and discussing it all together* (P7).” One woman had sought preconception counseling at the hospital through referral from her private psychiatrist. “*This [medication] was stopped with preconception counselling, a decision made at the time jointly with preconception counselling [at the hospital] and my private psychiatrist* (P4).” Patients were reassured when their usual treating doctor agreed with the specialist perinatal mental service recommendations, reinforcing a collaborative management approach and continuity of care. “*This was the advice with my private psychiatrist, and this was the same advice given by [perinatal psychiatrist] after I got pregnant* (P4).”

#### Challenges of managing medication

Most women in this sample presented for antenatal care with multiple prescribed psychotropic medication. Pregnancy was utilized as a motivator for change for some, with women able to reduce medications if clinically appropriate with the support and an acknowledgement of the difficulties. “*They kept emphasizing that I would be well supported, and it might be in mine and the baby’s best interests to try and wean off some of my medication so that’s why I eventually decided to try it. That was why I was successful in coming off two medications before I gave birth. I am still off those medications…* (P3).”

Ceasing medication was sometimes not a choice with adherence challenges compounded by the pregnancy. “*Occasionally I would forget to take things but that could be baby brain and being pregnant…In my pregnancy I really struggled with implementing stuff myself* (P1).” With regular antenatal appointments and concurrent psychiatric review, patients who struggled with medication adherence found routine monitoring beneficial. “*They would always ask me, like every appointment what I was taking…* (P1).”

### How information is given

#### Information delivery

A verbal exchange of information was reported regarding medication use during consultations, particularly for multiparous women who had discussed medication in previous pregnancies. “*I know all about it. Been through it heaps of times*’ (P2). Women who had been stable on medications for many years reported similar discussions ‘*all verbal, but to be honest it’s not like I started any new medications, so I already knew about the medications that I was on and how they affected me* (P3).”

Written information was appreciated; however, it was most beneficial when done as part of a discussion*.* “*I would prefer someone to sit down and talk than be given a pamphlet* (P8).” Written information allowed women to process it in their own time and gain clarification through further discussion with their treating clinician. “*[The psychiatrist] printed stuff out for me and would highlight the really important bits that I needed to look at. …would discuss it with me before they gave me the leaflets… That was really good* (P1)” and “*to have it all additionally down on paper to take home as well I found that easy for me* (P5).”

Being given ample opportunity to discuss treatments strengthened health literacy. “*[Psychiatrist was] amazing at talking these things through with me. And I think that if someone is talking things through and not just talking at you like, you have got to do this or you have got to do that but listening to where you are coming from and supporting you…* (P1*).*” Allowing questions to be asked in a supported environment was seen as beneficial. “*Being supportive, being listened to and answering all my questions, because I ask a lot of questions helped* (P7).”

Information delivered in early pregnancy needs to be re-evaluated when deteriorations in mental health require sudden changes at a later stage of the pregnancy “*I was on one medication and then it was changed to another at the last minute... You know, it’s a big thing - you’re thinking that you are on one medication… and then suddenly the advice is to go on this medication [lithium] and you can’t breastfeed* (P6).”

#### Communication breakdown

Some instances of communication breakdowns occurred due to cultural and language barriers. Even though several of the women in the study were deemed to speak English and interpreters were seen as not required, a lack of scrutiny occurred as to whether the information delivered had been adequately understood. “*…They gave me a pamphlet... they never discussed. They said …as this is the one you have been using, it was fine for pregnancy. … After they said it was ok to breastfed, but no other information given’* (P8) and *‘I never had an interpreter at any of the appointments* (P12).” These language barriers and cultural differences may have also impacted a woman’s sense of self-advocacy to ask for further clarification, “*[interviewer - Was this offered*?*] Not so much, but I didn’t really ask them to* (P12).”

### Breastfeeding dilemmas

#### Lithium use and breastfeeding choice

While most women’s choice to breastfeed was respected, it became more complex when women were treated with lithium. Woman also sought advice from other sources. “*I also saw a lactation consultant outside the clinic… she said she wouldn’t support me breastfeeding on lithium either* (P1)” and “*I always research on the internet* (P 11).” Women were offered options for medication changes to support the choice to breastfeed. “*They said the only way I could breastfeed was through valproate, but it doesn’t agree with my body…So I said no, I’d rather bottle feed* (P2)” and “*I really wanted to [breastfeed]…I was given information around the side effects of the medication on breast feeding. The fact that I couldn’t breastfeed on lithium, and because I was on the lower dose it was decided to come off lithium* (P7)*.*”

Women felt conflicting pressures regarding lithium. One woman expressed feeling pressure to cease lithium to breastfeed when she preferred formula feeding and attributed this to the “*pro-breastfeeding*” (P3) stance of the hospital. Another expressed feeling pressure to commence lithium treatment without proper acknowledgement of her intentions around breastfeeding. “*That was a bit of an issue deciding if I wanted to go on the lithium afterwards... this had been discussed previously before I got pregnant … but when going on lithium came up - it was all a bit of a last-minute thing…* (P6).” A shared decision approach enabled a compromise in this case. “*I gave the baby a couple of feeds before I went on lithium that night…I was happy enough to get those feeds in. That was planned and discussed* (P6).”

#### Autonomy regarding breastfeeding on medication

Autonomy over breast feeding choices was raised by several women in the study. After receiving information regarding transmission through breastmilk, they felt able to make informed decisions regarding medication changes. “*[The psychiatrist] had gone through the statistics of what could happen and how minor they actually were but left the decision up to me if I wanted to change the medication, but this was the most stable I had been in years* (P5)” and “*some babies get affected by it, some don’t. They were just - it’s up to you* (P10).”

One woman felt her decision regarding breastfeeding was not supported with some hospital staff unaware of the risks in women with SMI. She felt immense pressure to breastfeed against her initial plans, which ultimately had a negative impact on her mental state. “*I had initially decided not to breastfeed as I did it in my first pregnancy… the [midwife] encouraged me to breastfeed, whereas the psychiatrist didn’t...Unfortunately, I felt a little bit pressured to breastfeed and I became ill when on the ward after having the baby* (P11)” and “*…we were told different things in terms of breastfeeding. It would have been good to have more consistency with information* (P4).”

Women’s perspectives on breastfeeding influenced their treatment choices. Prioritizing mental health over breastfeeding for some women was seen as recognizing their own needs. “*To be honest there was other reasons as well [not to breastfeed] - bottle feeding was more convenient to me…part of my mental illness is that sleep is very crucial to being mentally healthy* (P3)” *and* “*it would mean that my husband could feed my son as well as I could. So even though I do the majority of all feeding it just allows me to have some time out and something he could help me with. So that was one of the main considerations for bottle feeding* (P3).” Some however enjoyed the positive mental health aspects of breastfeeding. “… *that emotional bonding, I really enjoy breastfeeding as I feel we have such a strong connection from that. It is an amazing connection. We both feel content when breastfeeding* (P5).”

## Discussion

Women with severe mental illness face difficult decisions when it comes to medication use due to the complex nature of their mental illness and the medication prescribed, alongside some uncertainty with the use of these medications in pregnancy and with breastfeeding. Findings from this qualitative study offer further understanding of patients’ experiences in decision-making about the use of psychotropic medication during pregnancy and breastfeeding. In our study, women reported factors that positively influenced the decision-making process around medication that included a shared decision-making model, having an established therapeutic relationship with a multidisciplinary team that viewed mental health as part of total patient care, collaboration including good communication involving external treating clinicians’ consistent information delivery, and a sense of agency and autonomy when making decisions for their treatment, especially regarding breastfeeding.

Decision-making among pregnant women in general, including those with anxiety and depression, is most strongly influenced by health practitioners, family, and the internet (Hameen-Anttila et al. 2013; Kothari et al. 2019). Health professionals need to discuss the information sources used, interpret this health information, and tailor it the specific needs of the individual as part of the collaborative decision-making (National Collaborating Centre for Primary 2009). While this is brought out to some extent in our interviews, the interaction with health practitioners for women living with SMI relies on delivering clear and targeted information and providing ongoing support.

Many factors in the health provider-patient interaction may influence the decision-making process of whether to continue or change antipsychotic medication, and giving adequate evidence-based information about the known safety profile may not be enough. Consistency of staff and collaboration, within a multidisciplinary team and with community health providers involved in their care, were reported in our interviews to support an environment of trust in which this could safely occur. The literature supports this approach, with trust seen as essential for willingness to follow health advice (Bakhireva et al. 2011) and continuity of care as an essential element in providing a trusting relationship in pregnancy care for women with SMI (Hauck et al. 2013). Women with epilepsy who are often on complex medication regimes had confidence in the information provided by their specialists, whose credibility appeared to have been enhanced by taking time to explain issues and answer questions as well as referring to the latest literature and consultation with other specialist colleagues (Widnes et al. 2012).

Health professionals are often regarded as a knowledgeable source of information but can sometimes be conflicting in their advice, especially as new evidence-based information becomes available. In fact, health professionals’ knowledge of medication safety in pregnancy and breastfeeding is widely variable (Csajka et al. 2014; Williams et al. 2020). Women who receive contradictory information can have a negative impact on their health (Baggley et al. 2004), and this may potentially be supported in the future using decision aids (Broughton et al. 2021). However, conflicting information can be given at any time across the continuum of care, as seen in several of our interviews, and is possibly best addressed through multidisciplinary education and clear documentation and planning. This is vital within a public health setting, as there remain challenges with staff flow and changes that can negatively affect the health provider-patient interaction.

Experiences reported by women on information provision from this research are supported by recent studies examining decision-making in the perinatal setting. A Cochrane review (Johnson and Sandford 2004) found that the combination of verbal and written health information enabled the provision of standardized care information to patients and family, which appears to improve knowledge and satisfaction. One important aspect of the process of informed consent for many is the finding that following any medical consultation, only 20–60% of the disclosed information is retained, and nearly half of which is wrong (Petersen et al. 2014), raising the need for the consultation process to be flexible with follow-up rather than a once-off discussion and tailoring the information to the needs of the individual. Both of these aspects were reported to be positive influences in our study.

Given the complexity of treatment and the risks involved in continuing or discontinuing pharmacological treatment in women with SMI, they require concise information presented in a timely manner that can be absorbed and reflected on. This is especially pertinent for women of culturally and linguistically diverse (CALD) backgrounds where written information followed by verbal discussion can enhance patient’s knowledge, satisfaction, and engagement (Tran and Castle 2009). Furthermore, it is important that we continually access the level of comprehension in women from CALD backgrounds and offer interpreters, even if they appear to speak adequate English, to improve overall health literacy. Though only two women in our sample were from a culturally diverse background, we found that health providers overestimated their comprehension of some of the issues around medication prescribing.

When considering engagement and collaboration, it is imperative that information be given in a sensitive, non-judgmental, and non-stigmatizing method that engages women with SMI and empowers them to make informed decisions. This was seen in our study, especially around breastfeeding, and how their preferences and mental health status impact medication choice, with women shifting their priorities regarding self-care and parenting and face potential stigma for doing so. The NICE guidelines (2014) recommend breastfeeding be encouraged in women with mental illness, and breastfeeding is an important concern for many who regard it as a central part of mothering and assists with infant attachment. With the public health emphasis on breastfeeding in terms of nutrition and welfare for babies, it is not surprising that many women have concerns and strong emotions about breastfeeding while on antipsychotic medication. Decision-making in regard to breastfeeding while on medication can be challenging, especially given reports of women receiving conflicting advice from health professionals (Baker et al. 2021) Some women in our study were able to self-advocate for their choices to breastfeed or formula feed where others felt rushed or pressured to decide about treatment.

Our study saw this for women who were prescribed lithium, with this treatment remaining controversial for breastfeeding women (Newmark et al. 2019) and not recommended based on risk benefit analysis (Galbally et al. 2018; Poels et al. 2018). Decision hesitancy regarding the need to continue treatment versus a desire to breastfeed was tempered in our participants by experience with medication and mental health stability. The concerns raised by women, however, do highlight that any change to long-standing medication should be discussed early, ideally in preconception counseling and repeated in pregnancy, with an individual’s choices remaining central to any decision-making (Galbally et al. 2018).

This qualitative study has enabled an exploration of the perspectives of women with SMI in pregnancy and breastfeeding, especially in relation to psychotropic medication use. This work adds to the literature in acknowledging these issues for women with SMI and gives greater understanding for health professionals to deliver improved services specific to their needs. A limitation of this study is that the sample is recruited from women who delivered in a specialized perinatal and obstetric multidisciplinary setting, potentially introducing a sampling bias toward those with severe illness. Despite this, the knowledge and challenges highlighted from these interviews are likely to be similar in other services and may be greater in areas with no or limited perinatal mental health services. A further limitation might include memory bias related to recollected events, increased by only interviewing the women and not the health workers. The experiences of women with bipolar disorders compared to those with schizophrenia, while not highlighted in our sample, would benefit from further exploration.

## Conclusion

Women with severe mental illness face unique challenges in the journey of motherhood, beginning at the point of preconception. Access to multidisciplinary care allows for greater collaboration and continuity of care and helps women in the decision-making process. Women in this group face difficult decisions that emphasize their dual role as both persons with a diagnosed mental illness but also new mothers, with many options causing conflict. It is the clinician’s role to provide women with comprehensible, consistent, and concise information, giving space for their voice to be heard, for them to be supported, and to guide them from a position of hesitancy to one of assurance.

## Supplementary Information

Below is the link to the electronic supplementary material.

Supplementary file1 (DOCX 19.5 KB)

## Data Availability

Excerpts from the qualitative data from the interviews have been included in the manuscript. All datasets generated during this current study are not publicly available, although quotes from interviews and demographic data sets are included in this article; further information may be available from the corresponding author on reasonable request.

## References

[CR1] Ando H, Cousins R, Young C (2014) Achieving saturation in thematic analysis: development and refinement of a codebook. *Compr Psychol* 3. 10.2466/03.Cp.3.4

[CR2] Bagadia A, Nanjundaswamy MH, Ganjekar S, Thippeswamy H, Desai G, Chandra PS (2020). Factors influencing decision-making around pregnancy among women with severe mental illness (SMI): A qualitative study. Int J Soc Psychiatry.

[CR3] Baggley A, Navioz Y, Maltepe C, Koren G, Einarson A (2004). Determinants of women’s decision making on whether to treat nausea and vomiting of pregnancy pharmacologically. J Midwifery Womens Health.

[CR4] Baker N, Potts L, Jennings S, Trevillion K, Howard LM (2021). Factors affecting infant feeding practices among women with severe mental illness. Front Glob Womens Health.

[CR5] Bakhireva LN, Young BN, Dalen J, Phelan ST, Rayburn WF (2011). Patient utilization of information sources about safety of medications during pregnancy. J Reprod Med.

[CR6] Banerjee D, Arasappa R, Chandra PS, Desai G (2021). “Hear me out”: experiences of women with severe mental illness with their healthcare providers in relation to motherhood. Asian J Psychiatr.

[CR7] Braun V, Clarke V (2006). Using thematic analysis in psychology. Qual Res Psychol.

[CR8] Broughton LC, Medlicott NJ, Smith AJ (2021). Effectiveness of patient decision aids in women considering psychotropic medication use during pregnancy: a literature review. Arch Womens Ment Health.

[CR9] Csajka C, Jaquet A, Winterfeld U, Meyer Y, Einarson A, Panchaud A (2014). Risk perception by healthcare professionals related to drug use during pregnancy: a Swiss survey. Swiss Med Wkly.

[CR10] Frayne J, Hauck Y, Sivakumar P, Nguyen T, Liira H, Morgan VA (2020). Nutritional status, food choices, barriers and facilitators to healthy nutrition in pregnant women with severe mental illness: a mixed methods approach. J Hum Nutr Diet.

[CR11] Frayne J, Nguyen T, Allen S, Hauck Y, Liira H, Vickery A (2019). Obstetric outcomes for women with severe mental illness: 10 years of experience in a tertiary multidisciplinary antenatal clinic. Arch Gynecol Obstet.

[CR12] Galbally M, Bergink V, Vigod SN, Buist A, Boyce P, Chandra P, Kohan R, Howard LM (2018). Breastfeeding and lithium: is breast always best?. Lancet Psychiatry.

[CR13] Hameen-Anttila K, Jyrkka J, Enlund H, Nordeng H, Lupattelli A, Kokki E (2013) Medicines information needs during pregnancy: a multinational comparison. *BMJ Open* 3(4). 10.1136/bmjopen-2013-002594 10.1136/bmjopen-2013-002594 PMC3641472 23624989

[CR14] Hauck Y, Allen S, Ronchi F, Faulkner D, Frayne J, Nguyen T (2013). Pregnancy experiences of Western Australian women attending a specialist childbirth and mental illness antenatal clinic. Health Care Women Int.

[CR15] Hippman C, Balneaves LG (2018). Women’s decision making about antidepressant use during pregnancy: a narrative review. Depress Anxiety.

[CR16] Howard LM, Kumar R, Thornicroft G (2001). Psychosocial characteristics and needs of mothers with psychotic disorders. Br J Psychiatry.

[CR17] Johnson A, Sandford J (2004). Written and verbal information versus verbal information only for patients being discharged from acute hospital settings to home: systematic review. Health Educ Res.

[CR18] Jones I, Chandra PS, Dazzan P, Howard LM (2014). Bipolar disorder, affective psychosis, and schizophrenia in pregnancy and the post-partum period. Lancet.

[CR19] Kim HG (2012). A piece of my mind. Drowning in plain sight. JAMA.

[CR20] Kothari A, de Laat J, Dulhunty JM, Bruxner G (2019). Perceptions of pregnant women regarding antidepressant and anxiolytic medication use during pregnancy. Australas Psychiatry.

[CR21] Morgan VA, McGrath JJ, Jablensky A, Badcock JC, Waterreus A, Bush R, Carr V, Castle D, Cohen M, Galletly C, Harvey C, Hocking B, McGorry P, Neil AL, Saw S, Shah S, Stain HJ, Mackinnon A (2014). Psychosis prevalence and physical, metabolic and cognitive co-morbidity: data from the second Australian national survey of psychosis. Psychol Med.

[CR22] National Collaborating Centre for Primary, C (2009). National Institute for Health and Clinical Excellence: guidance. *Medicines Adherence: Involving Patients in Decisions About Prescribed Medicines and Supporting Adherence*.

[CR23] Newmark RL, Bogen DL, Wisner KL, Isaac M, Ciolino JD, Clark CT (2019). Risk-benefit assessment of infant exposure to lithium through breast milk: a systematic review of the literature. Int Rev Psychiatry.

[CR24] NICE (2014). *Antenatal and postnatal mental health: the NICE Guideline on Clinical Management and Service Guidance*.

[CR25] Nicholson J (2014). Supporting mothers living with mental illnesses in recovery. *Motherhood, mental illness and recovery: stories of hope*.

[CR26] Petersen I, McCrea RL, Osborn DJP, Evans S, Pinfold V, Cowen PJ, Gilbert R, Nazareth I (2014). Discontinuation of antipsychotic medication in pregnancy: a cohort study. Schizophr Res.

[CR27] Poels EMP, Bijma HH, Galbally M, Bergink V (2018). Lithium during pregnancy and after delivery: a review. Int J Bipolar Disord.

[CR28] Shah N (2012). Mood disorder in the perinatal period. BMJ.

[CR29] Tran N, Castle D (2009). P.1.i.007 The effects of providing verbal and translated psychiatric medication information to non-English speaking (CALD) patients with a mental illness. Eur Neuropsychopharmacol.

[CR30] Widnes SF, Schjøtt J, Granas AG (2012). Risk perception and medicines information needs in pregnant women with epilepsy – a qualitative study. Seizure.

[CR31] Williams S, Bruxner G, Ballard E, Kothari A (2020). Prescribing antidepressants and anxiolytic medications to pregnant women: comparing perception of risk of foetal teratogenicity between Australian obstetricians and gynaecologists, speciality trainees and upskilled general practitioners. BMC Pregnancy Childbirth.

[CR32] Wisner KL, Zarin DA, Holmboe ES, Appelbaum PS, Gelenberg AJ, Leonard HL, Frank E (2000). Risk-benefit decision making for treatment of depression during pregnancy. Am J Psychiatr.

