# Supplementary material for: Experiences of decision making about psychotropic medication during pregnancy and breastfeeding in women living with severe mental illness: a qualitative study
Source: Arch Womens Ment Health. 2023 May 12;26(3):379–87. doi: 10.1007/s00737-023-01325-0 (PMC10191939; doi:10.1007/s00737-023-01325-0)
Supplement: Supplementary file 1 — Supplementary file1 (DOCX 19.5 KB) [file 737_2023_1325_MOESM1_ESM.docx]

**Postpartum qualitative interview guide**

Topic 1 Information

**Q1. Tell me what you thought of the information you received during your pregnancy?**

Areas to prompt:

- medication use specifically
- potential side effects
- supplements
- weight management/effects
- breastfeeding advice around illness and medication
- who delivered this information?

Topic 2 Barriers and facilitators

**Q2. What helped or hindered your experience with decision making with medication?**

Prompts for additional information:

- Give me an example of what worked for you?
- Give me an example of what didn’t work for you?
- What may have worked better for you? Why?
- What did not work well or made it more difficult in your situation?
- How would you prefer information to be given?

Topic 3 Support

**Q3. What did you think of the support you were given during this pregnancy?**

Prompts

- How did it help? Or not?
- What suggestions can you offer to help improve support?
- How did antenatal care support help?
- How important was it to see the same doctor, psychiatrist, midwife for these visits? Why?
- Did you breastfeed your baby and were you given any support? How did this affect things?
